# Lean and clinical risk management: an integrated implementation framework

**DOI:** 10.1108/IJHCQA-11-2025-0184

**Published:** 2026-06-18

**Authors:** Giulia Goretti, Martina Pisarra, Riccardo Speciale, Martina Lauria, Patrizia Meroni

**Affiliations:** Quality Department, IRCCS Humanitas Research Hospital, Rozzano, Italy; Department of Economics Management and Quantitative Methods (DEMM), Università Degli Studi di Milano, Milan, Italy

**Keywords:** Safety culture, Patient safety, Lean management, Process improvement, Clinical risk management

## Abstract

**Purpose:**

This study explores how the integration of lean management (LM) and clinical risk management (CRM) approaches enhances patient safety and operational efficiency by providing an integrated implementation framework.

**Design/methodology/approach:**

An empirical case study was conducted in a large academic hospital in Northern Italy, focussing on the hospital dietary process. A multiple–data-sources approach was employed for data collection, including participatory observations, surveys, structured interviews and secondary data.

**Findings:**

The integrated lean-clinical risk management approach increased incident reporting from 7 cases in 2020 to 30 in 2023, reflecting enhanced staff awareness and adverse event reduction. Diet prescriptions documented within 24 h rose from 71% to 99%, while kitchen order variations dropped from 52% to 2%. Patient satisfaction ratings increased by 11 percentage points. Operator time for diet management decreased significantly, from 750 to 47 h monthly.

**Practical implications:**

The study provides an integrated framework to support healthcare managers in integrating risk management and lean methodologies to boost clinical safety and process efficiency.

**Originality/value:**

The study fills a literature gap by providing empirical evidence of LM and CRM integration, offering actionable insights for sustainable healthcare quality improvement.

## 1. Introduction

A high-quality healthcare service is accessible, safe, timely, efficient, patient-centred and equitable. The World Health Organization (WHO) defines principles for healthcare organizations to provide high-quality healthcare service to patients (WHO, 2011), minimizing preventable harm and reducing risks during the delivery process (Stiefel and Nolan, 2013). Patient safety is one of the most important components of healthcare quality identified by the National Academy of Medicine and WHO (IOM, 2000), defined as “the prevention of errors and adverse effects to patients associated with health care”.

According to the Institute of Medicine (US) Committee, deaths due to medical errors are the 8th leading cause of death in the hospital. “The problem […] is that good people are working in bad systems that need to be made safe. The design of safe systems requires an understanding of the sources of errors and how to use safety design concepts to minimize these errors” (IOM, 2000).

Within the healthcare context, errors have two main causes, namely “omission”, which results from failing to take necessary actions, or “commission”, which occurs when an incorrect action is performed (Briner *et al.*, 2010). The most frequent errors are surgical, diagnostic, medication, device and equipment errors, infection, falls, communication, electronic records and hand-off errors (Rodziewicz *et al.*, 2024). Hospitals have thus developed methodologies to minimize errors. Among these, clinical risk management (CRM) has gained attraction within the academic and professional realms as a form of risk management that aims to identify risk situations for patients (Briner *et al.*, 2010) in clinical processes, and prevention actions for eliminating or controlling those risks (Crema and Verbano, 2015). This approach allows for improving quality in healthcare and, specifically, enhancing patient safety, reducing the costs of risks for healthcare providers (Walshe and Dineen, 1998). CRM initially involved a reactive approach, meaning that actions were taken primarily in response to adverse events or incidents after they occurred (Robinson, 1986). Then, it shifted to a proactive one (the creation of the Federal Patient Safety Act in the United States in 1983), focussing on anticipating and preventing potential risks before they result in harm, thus leading to the development of a culture of safety (IOM, 2000).

In recent years, other management approaches have been identified as prominent means to ensure quality in healthcare, thereby reducing errors. Such approaches include PDSA (Plan, Do, Study, Act), Lean management (LM), Six Sigma, total quality management (e.g. Varkey *et al.*, 2007; Benitez *et al.*, 2007; Kruskal *et al.*, 2012), among others. LM has significantly captured both scholars' and practitioners' attention for its potential to reduce waste, particularly clinical errors in the healthcare sector (Marsilio *et al.*, 2022). Coming from the Japanese manufacturing realm, it was first introduced into healthcare in the early 1990s (Womack *et al.*, 1990). It has been defined as an overall operational and management system that uses a culture of continuous improvement and focuses on meeting customer needs (i.e. the needs of patients, internal staff and the organization), improving quality while reducing waste (i.e. those activities that do not add value), optimizing organizational processes and patient flows and creating value (Womack and Jones, 1996). LM aims to eliminate waste (in Japanese, *Muda*), among which are errors; to reduce process variability (in Japanese, Mura), which creates stress in the process; to reduce overload (in Japanese, *Muri*), which creates high risks (Radnor *et al.*, 2012). Errors often arise from poorly designed processes and Lean Thinking addresses this by creating systems that minimize risks. Therefore, by systematically eliminating waste, reducing strainand levelling workload, Lean aims to design processes that minimize the chance for error, ensuring greater efficiency and effectiveness.

Recently, the literature on healthcare quality has stressed the potential of an integrated approach including CRM and LM to enhance patient safety, as these two approaches present a common ground in addressing risks and minimizing errors (Crema and Verbano, 2015). Although LM and CRM have been extensively studied, including in support services such as dietary and pharmacy services, and their conceptual connections have been acknowledged, the literature still lacks in-depth empirical studies documenting their practical integration, the interactions between the two approaches and the outcomes of an integrated process improvement strategy (Mendes and Franca, 2025). Studies have started to explore the complementary roles of CRM and LM in healthcare settings, but they often remain fragmented or focus predominantly on one approach over the other, failing to fully capture the synergistic effects of their integration. More in-depth research is required to systematically analyze how CRM and LM can be combined in practice, to identify best practices and critical success factors and to understand their joint impact on clinical safety outcomes. Building on this emerging research stream and addressing the call of prior studies (e.g. Crema and Verbano, 2015; Mendes and Franca, 2025), this paper aims at empirically investigating how an integrated LM and CRM approach enables to gain safer clinical processes and improves operational efficiency, by providing an integrated CRM-LM implementation framework.

To address this purpose, a case study (Yin, 2003) was conducted of the dietary process in a large academic hospital in Italy that combined clinical risk and LM approaches to improve patient safety and operational efficiency. This methodology allowed us to identify synergies between the two approaches and how they perform when implemented together. Dietary processes involve the prescription, preparation and delivery of patient meals, critical steps that directly impact patient safety and nutritional outcomes. Errors in these processes, such as incorrect meals or delays, can lead to adverse events and compromise care quality.

The paper is organized as follows. Section 2 presents CRM and LM features. Section 3 explains the methodology and the process before the intervention of LM and CRM. After the presentation of results in Section 4, Section 5 presents an integrated CRM-LM implementation framework.

## 2. Literature background

### 2.1 Clinical risk management

CRM is a form of risk management that focuses on preventing errors in clinical processes both directly and indirectly related to the patients. Derived from principles used in high-risk industries such as aviation, CRM promotes communication, teamwork and decision-making among healthcare professionals (Messano *et al.*, 2014). From a more practical point of view, CRM can be defined as guideline systems, protocols, steps, organizational and clinical procedures adopted by a hospital to reduce the probability that events and actions occur, as they might potentially produce negative or unexpected effects on the health of patients (Floreani, 2005). It adopts a multidisciplinary approach aimed at the identification, assessment and mitigation of risks inherent in the delivery of medical care. This approach employs a systematic methodology to prevent adverse events and to minimize the consequences of those that cannot be entirely avoided. In the domain of CRM, the analysis of adverse events is crucial for enhancing patient safety and healthcare outcomes (Vikan *et al.*, 2023). Various tools and methodologies have been developed to facilitate this analysis, aiding healthcare providers in identifying, evaluating and mitigating risks. Root Cause Analysis (RCA) and Failure Mode and Effects Analysis (FMEA) are essential for identifying vulnerabilities in healthcare systems and developing effective strategies to address them (Khon *et al.*, 2000). The RCA is a systematic process designed to explore the underlying factors contributing to an adverse event (National Patient Safety Foundation, 2015), while the FMEA helps proactively evaluate healthcare systems - such as clinical workflows, communication processes, equipment use and organizational protocols - to identify potential failures before they occur (Institute for Healthcare Improvement, 2020). Among others, these methodologies form the backbone of effective CRM strategies, promoting a culture of safety and continuous improvement within healthcare environments.

Then, Enterprise Risk Management was developed as a comprehensive approach that systematically identifies, assesses and manages risks in an organization to minimize impacts on financial stability, reputation and future growth. It extends beyond traditional risk management techniques by integrating risk management practices into all aspects of an organization's strategy and operations, focussing on the systematic use of tools and continuous improvement (Lucht, 2023). This holistic approach ensures that risk management is a continuous, strategically aligned process that involves all levels of the organization (COSO, 2017).

### 2.2 Lean healthcare management

LM emerged from the manufacturing industry, specifically in the Toyota Production System, with the aim of maximizing value by eliminating waste, improving efficiency and enhancing the quality of processes (Radnor *et al.*, 2012). This improvement approach is based on five principles, namely: (1) identifying value from the customers' perspective, by understanding their real needs and expectations; (2) mapping the value stream, to visualize all steps and eliminate non-value-adding activities; (3) identifying and improving the process flow, ensuring smooth and uninterrupted operations; (4) adopting a 'pull' logic, where production is driven by actual demand rather than forecasts; and (5) continuous improvement to achieve perfection, through ongoing evaluation and incremental changes (Womack and Jones, 1996).

The reduction of waste (or *Muda*) is the core purpose of Lean philosophy, which recognizes eight types: transportation, inventory, motion, waiting, overproduction, over-processing, defects and non-utilized talent. LM also focuses on reducing variability, known as Mura, which refers to “unevenness”. This highlights the importance of maintaining stable demand, which reduces fluctuations and helps create more efficient, standardized processes. Finally, *Muri*, meaning “excessive strain”, stresses the importance of providing workers with safe and sustainable working conditions to prevent physical strain and injuries (Crema and Verbano, 2017). This not only protects workers but also plays a significant role in reducing absenteeism and maintaining long-term productivity (Katayama, 2017).

Lean was applied to the healthcare domain since 2000. Over the past two decades, the implementation of Lean principles in healthcare has shown considerable potential in enhancing health system performance, improving productivity, flexibility, responsiveness, efficiency, process capability and quality, while also contributing positively to patient safety and mortality outcomes (Tiso *et al.*, 2021).

**Figure 1:**
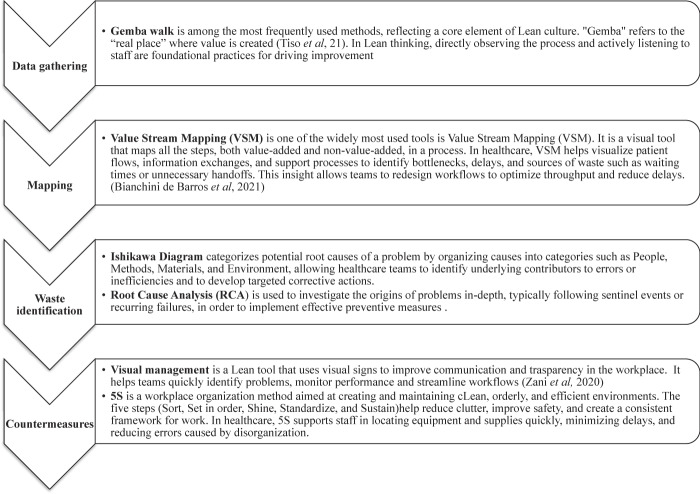
Examples of LM tools. Source: Authors' own work

Beyond a philosophy, LM is also a “tool-based approach” as it exploits several tools and technologies to reduce waste and improve processes, such as Value Stream Map (VSM), Gemba Walk, RCA, etc. Depending on the implementation phase and activities, different LM tools are adopted as reported in Figure 1.

Collectively, these tools and methodologies constitute a comprehensive toolkit that enables healthcare organizations to streamline processing times, reduce operational costs and alleviate workforce burdens, thereby improving overall organizational efficiency and patient safety. The transition towards Lean thinking necessitates the development of an organizational culture that empowers and motivates employees to actively participate in continuous improvement efforts (Hardcopf and Shah, 2014). A culture of continuous improvement encapsulates the attitudes and behaviours that foster a sustained commitment to excellence (Endalamaw *et al.*, 2024).

While some organizations focus on minimizing failures, within LM, failures are perceived as opportunities for learning and advancement. All personnel embrace a systemic approach to identifying process errors without attributing blame to individuals. Every member of the organization, including frontline staff, management and governance bodies, assumes a vital role in cultivating and sustaining this culture of improvement. A critical component of this commitment is the empowerment of employees to halt processes upon detecting defects, consistent with the principle of *jidoka*.

Central to LM is the concept of quality at the source, which underscores the imperative to prevent or immediately rectify errors at their point of origin, a principle often operationalized through tools like the Patient Alert System (*Andon Cord*), enabling frontline staff to signal problems in real time and trigger immediate response (Furman and Caplan, 2007). This principle is reinforced through fostering a culture of vigilance and investing in error-proofing mechanisms. Among these, *poka-yoke* refers to the design of processes incorporating inherent safeguards that prevent incorrect actions or render errors immediately apparent. Such mechanisms function as fail-safes that mitigate the incidence of mistakes, thereby enhancing process reliability and quality (Crema and Verbano, 2015).

### 2.3 Comparing CRM and LM approaches

Tiso *et al.* (2025) integrated the systematic, data-driven framework of the Define, Measure, Analyze, Improve, Control (DMAIC) cycle (Antony *et al*., 2017; Antony and Sony, 2020) with a participatory and iterative approach. This methodology was used generically to outline the various phases of a CRM-LM implementation process.

Their approach, applied to process improvement within healthcare contexts, leverages DMAIC to structure each phase (from problem definition and measurement through to implementation and control) while actively engaging stakeholders in a collaborative and iterative change process. This integration facilitates both rigorous analysis and inclusive participation, enhancing the effectiveness and sustainability of healthcare process improvements.

Building on this foundation, our model adapts the DMAIC structure in Table 1 to compare how Risk Management and LM identify errors or waste, implement corrective actions and sustain improvements over time. While Crema and Verbano focus on Lean-based interventions, our framework extends the application of DMAIC to include tools and strategies from both methodologies, offering a broader lens through which to enhance patient safety and quality of care.

**Table 1:** DMAIC cycle: LM VS RM

DMAIC phase	Risk management	Lean management
Define	Incident reporting, Morbidity and Mortality, Trigger Tools	Patient safety alert system, Gemba walk
Measure	Electronic medical records (EMRs)	Interviews, Data Management System
	Key performance indicators (KPIs)	
Analyze	FMEA, RCA, Audit/SEA	RCA, Value Stream Mapping (VSM)
Improve	Procedures	Standard work, Poka-yoke, Visual management, 5S
Control	Incident reporting, Clinical KPI monitoring	Dashboard, Audit plans, A3

**Source(s):** Authors' own work, Adapted from Crema and Verbano (2015)

In the “Define phase”, Risk Management utilizes structured methods such as Incident reporting (Kumah *et al.*, 2024), Morbidity and Mortality (M&M) reviews (Soltani *et al.*, 2021) and the Trigger.

Tools (Classen *et al.*, 2008) to identify adverse events and system vulnerabilities. These practices emphasize a retrospective analysis of clinical events and are instrumental in initiating safety improvement cycles (Leistikow *et al.*, 2017). LM, in contrast, focuses on real-time observation and frontline engagement through tools such as Gemba walks (McClam Liebengood *et al.*, 2013) and Patient safety alert systems (Furman and Caplan, 2007), aligning with its emphasis on value creation and waste reduction.

The “Measure phase” further differentiates the two approaches. CRM relies heavily on quantitative data sources, including electronic medical record (EMR) and prompt risk analyses, while LM prioritizes qualitative and contextual data collection through interviews and the use of digital Data Management Systems to identify process inefficiencies.

During the “Analyze phase”, both approaches adopt RCA (Singh *et al.*, 2025), but diverge in supplementary tools: Risk Management incorporates FMEA (El-Awady, 2023) and significant event analysis (Hut-Mossel *et al.*, 2021), whereas LM leverages VSM (Marin-Garcia *et al.*, 2021) to visualize and analyze the flow of activities and detect sources of non-value-adding steps (Kanabar *et al.*, 2024).

In the “Improve phase”, LM stands out for its structured improvement tools - such as standard work protocols (Kupec *et al.*, 2022), Poka-yoke (error proofing) (Grout, 2006), Visual management (Zani *et al.*, 2020) and the 5S methodology - which have demonstrated positive impacts in reducing inefficiencies and enhancing workplace organization in healthcare settings (Kanabar *et al.*, 2024). Risk Management, meanwhile, tends to focus on the formalization and revision of clinical procedures based on analytical findings.

Lastly, the “Control phase” reflects the monitoring strategies employed to sustain changes. Risk management emphasizes clinical Key Performance Indicators (KPIs) and ongoing incident reporting, while LM combines process KPIs, A3 reports (Ghosh, 2012) and satisfaction structured interviews to reinforce continuous learning and adaptive performance monitoring (Leistikow *et al.*, 2017).

This juxtaposition underscores how both methodologies, though distinct in orientation, can be complementary when applied within a shared DMAIC framework. Risk Management offers rigour and compliance with a focus on patient safety, whereas LM contributes agility and cultural change toward continuous improvement. Their integration provides a robust foundation for organizational learning and performance excellence in healthcare systems (Tiso *et al.*, 2025; Kanabar *et al.*, 2024).

## 3. Research design

### 3.1 Method

The paper employed an exploratory case-study methodology (Yin, 2003) in order to explore synergies between LM and CRM and their performance when implemented together. This approach allowed us to analyze the revision of a dietary process conducted in a large hospital in order to improve quality and patient safety, as well as operational efficiency.

### 3.2 Study setting

The study was conducted in an academic and research hospital located in Northern Italy, where an organizational strategy systematically integrates LM and CRM practices to optimize care pathways, streamline administrative processes and enhance both professional and patient safety. Since 2022, the teams responsible for LM and CRM have been integrated into a single organizational unit, fostering closer collaboration. These teams now hold regular meetings to address emerging challenges and evaluate improvements. The hospital's adoption of a “no-blame culture” and its overarching commitment to preventing future errors have been central to this approach. Subsequently, the two teams focused on analyzing various adverse events to identify the most effective strategies for mitigating the risk of future incidents.

### 3.3 The hospital dietary process

The dietary process refers to the systematic management of patient nutrition, including meal planning, preparation and delivery, tailored to meet specific medical and nutritional requirements. It ensures that patients receive appropriate diets based on their health conditions, allergies and therapeutic needs, playing a crucial role in patient recovery and overall well-being.

In the hospital, the dietary process involved approximately 1,200 meals per day, catering to 31 distinct dietary plans. This service covered 20 departments and over 30 clinical units.

Dietary prescriptions were issued by doctors through the hospital EMR system, which allowed them to choose from 31 different diet plans. While the software algorithm included a mechanism for excluding specific foods based on allergies and intolerances, healthcare professionals typically noted these in a free-text field rather than using a coded format. This practice prevented the automatic exclusion of allergenic foods.

Technically, dietary prescriptions were required to be made during the patient's clinical assessment. However, entries could be made either in the EMR or directly in the kitchen management system on a daily basis, leading to inconsistencies and confusion.

Nurses and Health Care Assistants (HCAs) played a crucial role in booking meals and verifying clinical prescriptions across multiple systems. They spent approximately one hour per department each day updating the kitchen management system, which was used to prepare meals.

Every day, staff members visited wards to collect patients' meal preferences based on their prescribed diets in the electronic system. However, if the system was not updated in time, they were unable to record preferences, or existing choices were lost when the kitchen management system was refreshed. This inefficiency negatively impacted the patient experience.

Additionally, the system was not perceived as intuitive or user-friendly.

**Figure 2:**
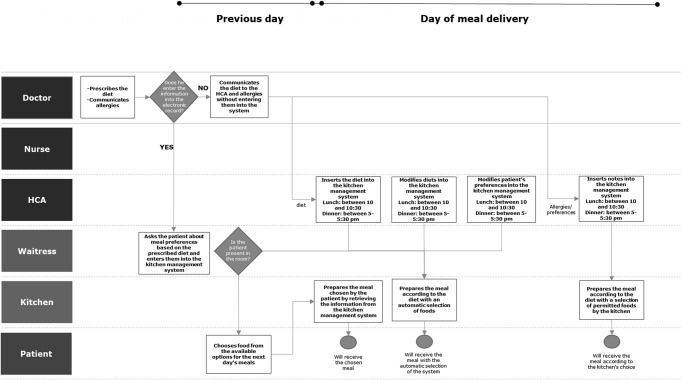
Process “as is”. Source: Authors' own work

Process “as-is” is described in Figure 2.

### 3.4 Data collection and analysis

Data collection involved multiple sources (Hopper and Hoque, 2006) and it was facilitated by the affiliation of two of the authors with the LM-CRM hospital team.

Firstly, archival analyses were conducted of hospital procedures, mapping processes, activities and actors involved. Secondary data analyses of hospital databases, such as electronic medical record (EMR) and kitchen management software, were performed to monitor operational performance and safety outcomes.

Pre-intervention data and percentage values were collected through a one-off, manual data collection activity conducted prior to April 2023, corresponding to the beginning of the improvement project. As historical data were not systematically recorded in digital form at that time, baseline information was reconstructed through manual extraction from operational records, internal reports and direct verification with staff involved in dietary service activities, with data primarily collected in textual note fields. This manual baseline assessment provided a reference point for interpreting post-intervention performance indicators.

Although the hospital was equipped with an integrated monitoring and business intelligence system, designed to collect and harmonize data from multiple institutional repositories, including the EMR and the dietary management platform (Kitchen Management System), this system was not actively used before the implementation of the project.

This infrastructure enables centralized data collection from clinical and operational sources, supporting cross-departmental analysis and continuous monitoring of healthcare processes, including dietary service workflows.

Post-intervention patient-related dietary data were collected from the EMR and from the dietary management platform (Kitchen Management System), which consolidates meal orders, diet types and related operational information. These data were subsequently integrated into the hospital's data warehouse, enabling structured storage and aggregation across different units.

**Figure 3:**
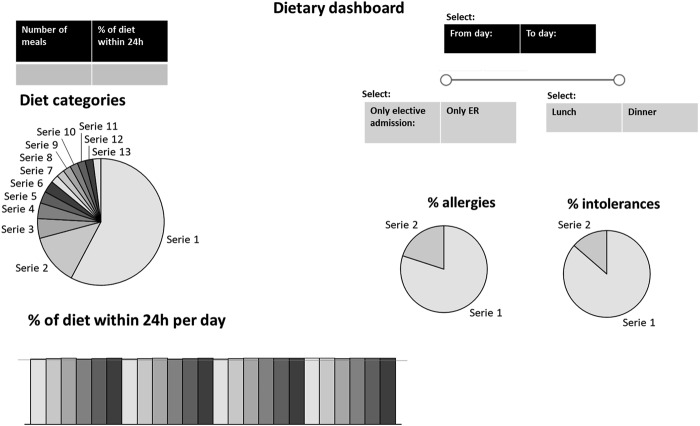
Dietary dashboard structure. Source: Authors' own work

For analysis and visualization, the extracted data were imported into Power Business Intelligence, where a dedicated dashboard was created to monitor KPIs, including the number of patients served, potential meals, trays delivered, diets prepared within 24 h and other relevant operational metrics (Figure 3). This dashboard facilitated real-time monitoring of dietary processes and supported quantitative assessment of process improvements. All data handling followed hospital data governance policies to ensure patient privacy and compliance with regulatory standards.

Participatory workshops and field observations engaged a cross-functional team to collaboratively analyze and improve patient meal delivery processes.

Incident reporting systems and ongoing data monitoring ensured continuous evaluation of adverse events. This multi-method approach enabled robust triangulation of diverse data sources, supporting a comprehensive understanding of hospital processes and informing effective interventions.

Table 2 provides a comprehensive overview of the case phases, aligned with the structured problem-solving methodology outlined in the DMAIC cycle, which is widely recognized as a core methodological component of the Lean Six Sigma approach, rather than a standalone improvement tool (Antony *et al*., 2017; Antony and Sony, 2020).

**Table 2:** DMAIC overview of the case phases

	DMAIC	Method applied	Activities
Define	1. Problem definition	Incident reporting	Risk and Lean team analyzing adverse events
	2. Workgroup determination	Interviews	Data were collected through the following methods Semi-structured interviews with members of the multidisciplinary team involved in the dietary planning process to gain in-depth insights into operational challenges and collaboration Participatory workshops and observations to engage frontline staff and management in analyzing and improving patient meal delivery. These sessions included process mapping activities to visually outline and better understand current workflows and identify areas for improvement
	2.1 Context description and process mapping	Workshop	
Measure	3. Data collection	Gemba walk	The team analyzed data from the electronic medical record (EMR) and data management system, kitchen management system, to create a shared understanding of the issue
		Data analysis	
Analyse	4. Analysis	Workshop	- September 2021 – RCA with the workgroup
Improve	5. Improvement proposal	Workshop	- October 2021 – Description by the project team of a standardized poka-yoke process involving information systems - December 9, 2021 – Proposal presented to hospital management Process paused due to the COVID-19 pandemic - September 2022 – Project approval, status unchanged
	6. Implementation plan (GANTT)	Workshop	- October 2022: Modifications to the IT system - November 2022: Changes to the kitchen management software - December 2022: Training for nursing, medical and kitchen staff - January–February 2023: Pilot implementation on one inpatient ward - March 2023: New Dietary Procedure - March 2023: Hospital-wide training through workshops and brief educational sessions - April 2023: Project launch, status unchanged
Control	7. Data analysis and control	Interview	- July 2023: Internal structured interviews conducted among physicians and nurses/healthcare assistants - November 2023: Second structured interview administered - Monthly patient experience structured interview - Dashboard generated through daily updated electronic reports - Incident reporting system implemented
		Data analysis	
	8. Follow Up	Data analysis	Dashboard
		Workshop	Audit plan

**Source(s):** Authors' own work

Within the Lean Six Sigma framework, the DMAIC framework serves as a systematic and visual management tool to support process improvement, waste reduction and integrated quality management by guiding problem definition, RCA and solution development. Each phase details the methods applied and the key activities carried out. The process begins with problem identification and the formation of a dedicated workgroup, followed by context analysis and process mapping. The subsequent phases involve in-depth analysis, development of improvement proposals and planning for implementation. Continuous data analysis, along with monitoring and follow-up actions, is conducted to ensure the sustainability and effectiveness of the improvements. This structured Lean Six Sigma-based DMAIC approach enables clear communication, effective collaboration among multidisciplinary teams and robust project management throughout the entire improvement cycle.

## 4. Results

The following paragraphs report both the integrative approach based on CRM and LM used to revise and improve the dietary process and its impacts.

The system improved timely diet prescriptions in the EMR and reduced kitchen order changes. Patient satisfaction increased, while operator time for diet management dropped significantly. Enhanced staff awareness led to more error reporting, helping prevent patient harm and achieving zero harm events.

### 4.1 The CRM-LM approach

The project involved a cross-functional team including a Risk Manager, a Lean Manager, members of the risk management team (physicians and nurses), nurses, healthcare assistants, kitchen staff, general services staff, the nutrition team and physicians.

Collaborative efforts led to the creation of VSM exercises tailored to different stages of the process, with direct input from nurses, healthcare assistants, physicians, nutrition team members and the risk management and Lean teams. These individual VSMs were subsequently consolidated into a single unified model during a joint meeting.

First, a Gemba walk was conducted to observe the workflow and collect detailed data. Based on the insights gathered, the team completed a comprehensive RCA, establishing clear cause-and-effect relationships to guide improvement planning.

Following this, a thorough clinical review was integrated into a redesigned workflow applying the poka-yoke principle. This systemic approach involved separating, rearranging, streamlining, standardizing and sustaining tasks and actions to minimize errors and enhance process reliability.

Currently, across 20 inpatient wards, the process encompasses all phases from prescription, meal preference selection, dietary modifications, kitchen preparation, to meal delivery to patients.

#### 4.1.1 Influential factors and impacts

**Figure 4:**
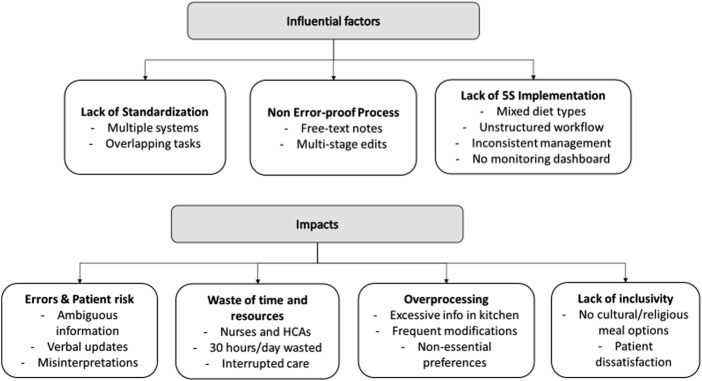
Influential factors and impacts. Source: Authors' own work

The main causes of errors identified in the process stemmed from several key issues (See Figure 4). Firstly, the lack of standardization led to the use of multiple systems and overlapping tasks among healthcare professionals, which resulted in inconsistencies and inefficiencies. Additionally, the process was not error-proof: the ability to enter free-text notes and modify information at different stages increased the risk of errors and miscommunication. Lastly, the insufficient implementation of the 5S methodology contributed to organizational disorder. Specifically, clinical diets were mixed with exclusion-based diets (Sort), the workflow was poorly structured (Set in Order), prescription management and modifications were handled inconsistently without standardization (Standardize) and the absence of a centralized dashboard hindered proper process monitoring and control (Sustain).

These mistakes caused four main effects. First, patient safety was compromised due to ambiguous and frequently changed information; many updates were communicated verbally, increasing the potential for misunderstanding and errors. Second, there was a significant waste of time and resources: nurses and healthcare assistants had to interrupt patient care to manage dietary prescriptions, spending on average over one hour per day per hospital unit, amounting to approximately 20 h daily across the entire hospital solely organizing patient meals. Third, over processing occurred as the hospital kitchen became overloaded with excessive information, including frequent diet modifications, allergy and intolerance records and non-essential patient preferences based solely on taste, which led to inefficiencies. Finally, a lack of inclusivity was observed, as the meal service did not fully accommodate patients' cultural and religious dietary preferences. The absence of diverse meal options left some patients underserved, contributing to dissatisfaction.

#### 4.1.2 Clinical review

The revision process centred on optimizing diet prescriptions by prioritizing value and aligning dietary options with the diverse clinical pathways encountered by patients. Restricted foods were managed through standardized exclusions applied uniformly across all diet types. Consequently, the number of diet categories was streamlined from 31 to 13, enhancing efficiency and clarity within the dietetic workflow.

Standardized filters were integrated to account for portion sizes, intolerances, allergies, clinical requirements, religious practices and texture modifications. This framework substantially expanded the potential combinations of diet options, thereby enabling a high degree of personalization tailored to individual patient needs.

An additional enhancement in the revised diet protocols was the introduction of snack options, encompassing sweet, savoury and soft textures. These snacks are designed to facilitate patients' reintroduction to oral intake following surgical interventions or medical procedures.

#### 4.1.3 New process features

**Figure 5:**
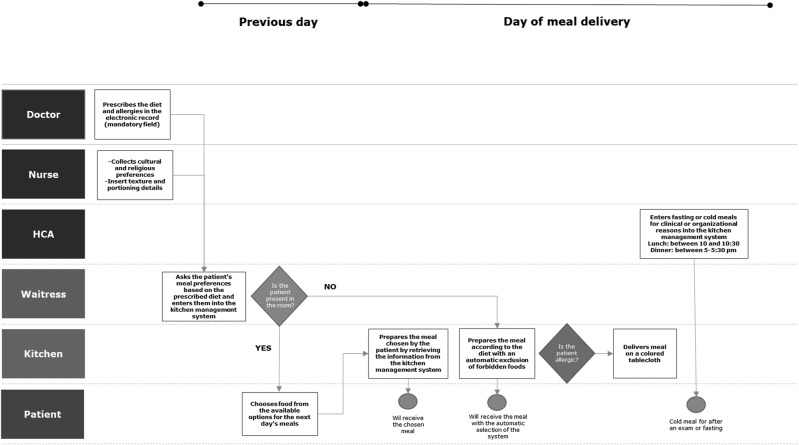
The new process. Source: Authors' own work

A standardized and mandatory workflow (See Figure 5) was implemented whereby diet prescriptions became an integral section within the EMR system. Clinicians prescribe clinical diets according to the patient's clinical pathway and can modify prescriptions within the EMR as necessary. Allergies, intolerances and specific nutritional requirements are entered and managed through an automated exclusion system. Nurses record information on food consistency, portion sizes and religious or cultural dietary needs in a designated section of the EMR. Importantly, HCAs and nurses are restricted from modifying physician-prescribed diets within the kitchen management system. The information flow to the kitchen remains fixed after initial entry, with no subsequent alterations permitted. Patient food preferences collected by staff are preserved and not overwritten. The system excludes free-text fields for HCAs, nurses, or physicians, ensuring that all clinical records are precise and unambiguous. This strict traceability, combined with the elimination of free-text inputs, facilitates efficient report generation and improves the identification of allergies and specific dietary needs during meal preparation.

To enhance patient safety and dietary customization further, a Lean visualization technique was introduced to signal allergies. A distinctive blue tray mat was employed for patients with allergies, serving as an immediate and clear visual indicator of their specific dietary requirements.

### 4.2 Impacts

To complement the observational and dashboard data, a structured interview aimed to capture participants' perceptions of the implemented LM - CRM improvements was administered (See Table 3).

**Table 3:** Overview of participants in structured interviews

Role	Number of participants
Nursing coordinators	13
Nurses	62
Healthcare assistants	47
Total	*122*

**Source(s):** Authors' own work

The improvement approach based on CRM and LM led to several benefits for patients (See Table 4). The proportion of diet prescriptions documented in the clinical record within 24 h increased markedly from 71% to 99% (achieving 100% within 48 h, largely due to emergency room-ER- procedures). This advancement enhances traceability, reduces potential dietary errors and strengthens accountability in diet prescription. Correspondingly, 61% of nursing coordinators agreed that patient safety levels improved.

**Table 4:** Project's impacts

Area	Indicator/Measure	Before April 2023	After April 2023	Comments
Diet prescription	% of diet prescriptions documented in EMR within 24 h	71%	99%	100% within 48 h due to ER procedures; improves traceability and reduces dietary errors
Patient safety	Nursing coordinators acknowledging improved safety	–	61%	Positive feedback on patient safety impact
Kitchen variations	% of variations requested to the kitchen collected by e-mail	52%	2%	Variations now limited to immediate clinical/organizational adjustments
Patient satisfaction	% of patients rating satisfaction 8, 9, or 10	59%	70%	Increase attributed to improved diet management and engagement
Organizational efficiency	Operator working hours per month	750 h	47 h	Confirmed by 74% of HCAs reporting decreased documentation time
Quality culture	Number of incident reports related to dietetic errors	7	30	Increased error detectability; no incidents resulted in harm due to effective risk barrier

**Source(s):** Authors' own work

Furthermore, variations requested by the kitchen decreased substantially from 52% to 2%, now limited to immediate clinical or organizational adjustments. Patient preferences collected by staff were always confirmed without modifications. These advancements also contributed to higher patient satisfaction, with ratings of 8, 9, or 10 increasing from 59% to 70%, based on data collected through weekly questionnaires administered to all hospitalized patients regarding the quality of meals.

From an organizational standpoint, the system demonstrated enhanced efficiency in dietetic management, reducing operator working hours from 750 to 47 h per month. This efficiency gain was corroborated by 74% of healthcare assistants who agreed that time dedicated to dietetic documentation had decreased.

Staff awareness and involvement during analysis phases led to increased error detectability, with incident reports rising from 7 in 2020 to 30 in 2023. Importantly, none of these incidents resulted in harm, indicating that the implemented risk-reduction barriers were effective.

**Figure 6:**
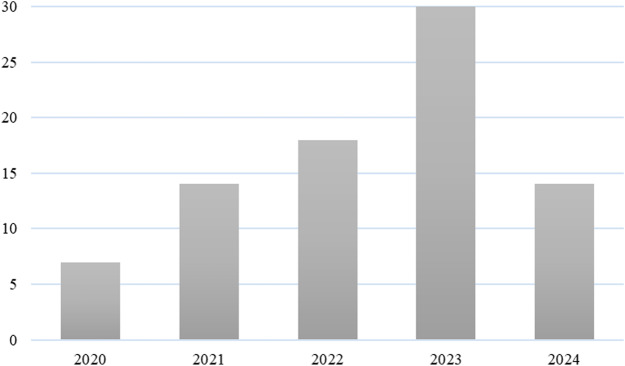
Number of Incident reporting cases from 2020 to 2024. Source: Authors' own work

Figure 6 illustrates the trend in incident reporting from 2020 to 2024, showing an initial rise in 2022 corresponding with staff engagement and system rollout, followed by a reduction in 2024 attributable to enhanced error-proofing measures.

**Figure 7:**
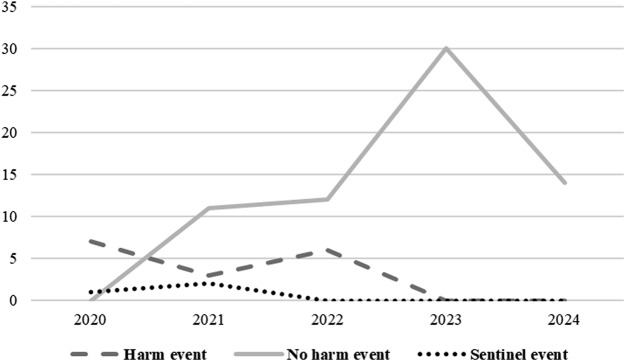
Number of patient safety events by type from 2020 to 2024. Source: Authors' own work

The new process has successfully eliminated patient harm events by enabling timely interception of potential errors, in contrast to previous occurrences where errors were often detected only after causing minor or serious harm (sentinel events), as depicted in Figure 7.

## 5. Discussion

The paper explored how LM and CRM can be jointly applied to gain safer clinical processes. The findings show how an integrated LM and CRM approach was implemented to improve a dietary process, leading to higher patient safety, operational efficiency and healthcare operators' wellbeing.

Specifically, the case analysis allowed the construction of a process with greater barriers in defence of the patient. This improves not only safety but also the value-added time of the involved operators.

This research responds to the call by Crema and Verbano (2015), as well as by Mendes and Franca (2025), for more empirical evidence on the implementation of a joint approach. The study adds to the existing literature on how LM can provide solutions to address risk management issues (Mendes and Franca, 2025) by not only providing an empirical implementation of an integrated approach but also showing the results stemming from such implementation.

**Figure 8:**
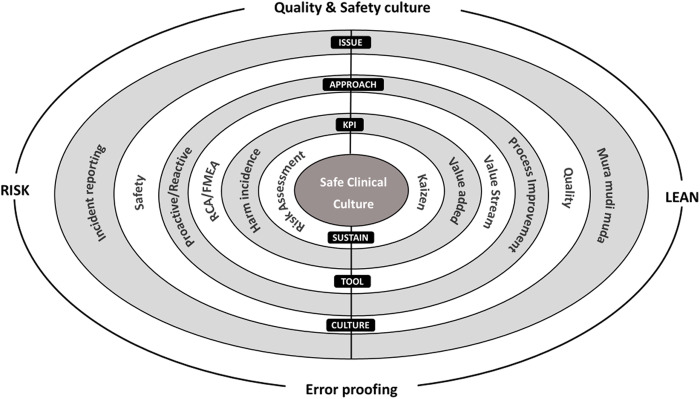
Framework lean and risk, an integrated approach combining lean and risk management principles. Source: Authors' own work

While the literature has extensively examined LM and CRM frameworks, addressing both their conceptual foundations and technical tools (Marsilio *et al.*, 2022), this study contributes to the field by proposing the first integrated LM–CRM framework (Figure 8). The framework enables a strategic alignment between LM and CRM principles, demonstrating that their integration is not only necessary but also important for the success of process improvement initiatives in clinical pathways, as well as in support service pathways. The research provides a first empirical validation within support services, specifically dietary processes, which the literature have investigated using CRM and LM as separate improvement approaches (LM or CRM) (Paul Teeling *et al.*, 2019).

Although the framework is developed on the basis of a dietary process case, it can be adapted to other hospital settings, such as pharmaceutical prescribing processes. Accordingly, the framework is designed to be scalable and flexible, allowing organizations to “zoom in” or “zoom out” by selecting specific technical tools while maintaining a consistent overarching strategy that integrates LM and CRM principles for mutual support.

CRM and LM together help reduce errors, since the former focuses on adverse events using *incident reporting*, while the latter targets the reduction of waste (*muda*), variability (*mura*) and overload (*muri*). Together, they provide a structured methodology to address process errors comprehensively. A shared *culture of quality and safety* underpins both approaches, acting as the foundation for *sustainable improvement*.

Data were analyzed jointly, enabling the integration of *RCA*, a *reactive* tool, with *FMEA*, a *proactive* risk management strategy. This dual approach strengthens the robustness of the analysis. A *VSM* was also designed, incorporating error-proofing mechanisms (such as alerts for allergies or restrictions on diet modifications), thereby aligning process design with safety goals. Value was defined from an evidence-based clinical practice perspective, ensuring that improvements are anchored in patient-centred care.

The iterative nature of the process was reflected in the development of a continuous improvement report, with a documented increase in incident reporting during the implementation phase. This not only signifies growing engagement but also enhanced awareness and responsiveness within the organization. The results, namely, the reduction of *harmful errors* and elimination of *non–value-added activities*, enabled a truly in-depth analysis of each event, addressing both patient safety concerns and organizational-process inefficiencies.

The integration of these elements leads to what Lean defines as *Kaizen*, a philosophy of continuous, incremental improvement involving all levels of the organization. When combined with a rigorous and systematic *Risk Assessment* process, the outcome is a comprehensive framework that supports proactive, data-driven decision-making.

Ultimately, this synergy fosters the development of a *Safe Clinical Culture*, where safety, accountability, flow and effectiveness become guiding principles. Such a culture emerges precisely from the integration of Kaizen and Risk Assessment, encompassing all the components analyzed throughout this framework, from RCA to value stream design and from error prevention to staff engagement. This model provides healthcare organizations with a structured and replicable pathway to achieve both clinical excellence and operational resilience.

Although exploratory in nature, this study is limited to a single case, which limits the generalizability of the findings. Future research could thus test the model in other healthcare contexts or in other improvement processes. Moreover, despite triangulation was used, due to the real-world nature of the intervention, comprehensive pre-intervention quantitative data were not available in the hospital databases for direct comparison with post-intervention outcomes.

Due to the absence of structured and continuous pre-intervention time-series data, it was not possible to conduct formal trend analyses or variability assessments (e.g. standard deviation or statistical process control). However, baseline process conditions were not entirely unknown: pre-intervention information was reconstructed through manual data extraction, document review and direct consultation with staff involved in dietary service operations. This qualitative and descriptive baseline assessment informed the design of the intervention and provided a reference for interpreting post-intervention quantitative indicators. Further researches should deepen the outcomes of the integrated approach and use different data collection methodologies such as surveys to provide data for more accurate statistical analyses, including inferential analyses.

## 6. Practical implications

The study demonstrates that an integrated CRM–LM approach can successfully lead to process improvements, enhancing both patient satisfaction and organizational wellbeing. It also provides a conceptual framework to implement such an integrated approach. Beyond its contributions to the LM and CRM literature, the evidence has practical relevance for healthcare organization.

From a practice perspective, the proposed framework can support healthcare quality managers in designing improvement initiatives starting from risk management-related issues, as well as risk managers who are required to intervene when operational problems may compromise patient safety. The study highlights that CRM and LM should be regarded as complementary, rather than separate, approaches and therefore integrated within hospital settings to be effective.

From a teaching and training perspective, the process mapping and improvement strategies discussed in this study can be used to inform staff training programs and to serve as illustrative case studies in courses on healthcare process design, quality improvement and patient safety.

From a policy and governance perspective, the findings suggest that integrated CRM-LM interventions may inform managerial and organizational policies aimed at improving healthcare quality and safety.

However, the implications for practice, teaching and policy should be considered provisional and contingent upon further evaluation. Managers and policymakers should implement similar interventions with caution, ensuring careful monitoring, context-specific adaptation and systematic evaluation of outcomes. Finally, the study points to avenues for future research, including longitudinal analyses with more comprehensive data collection and comparative studies across multiple hospital departments.
